# Supplementary material for: The causal relationship between the human gut microbiota and pyogenic arthritis: a Mendelian randomization study
Source: Front Cell Infect Microbiol. 2024 Nov 26;14:1452480. doi: 10.3389/fcimb.2024.1452480 (PMC11629706; doi:10.3389/fcimb.2024.1452480)
Supplement: Supplementary file 1 [file DataSheet1.zip › mendelian test/ebi-a-GCST90027508.csv_leave_one_out.pdf]

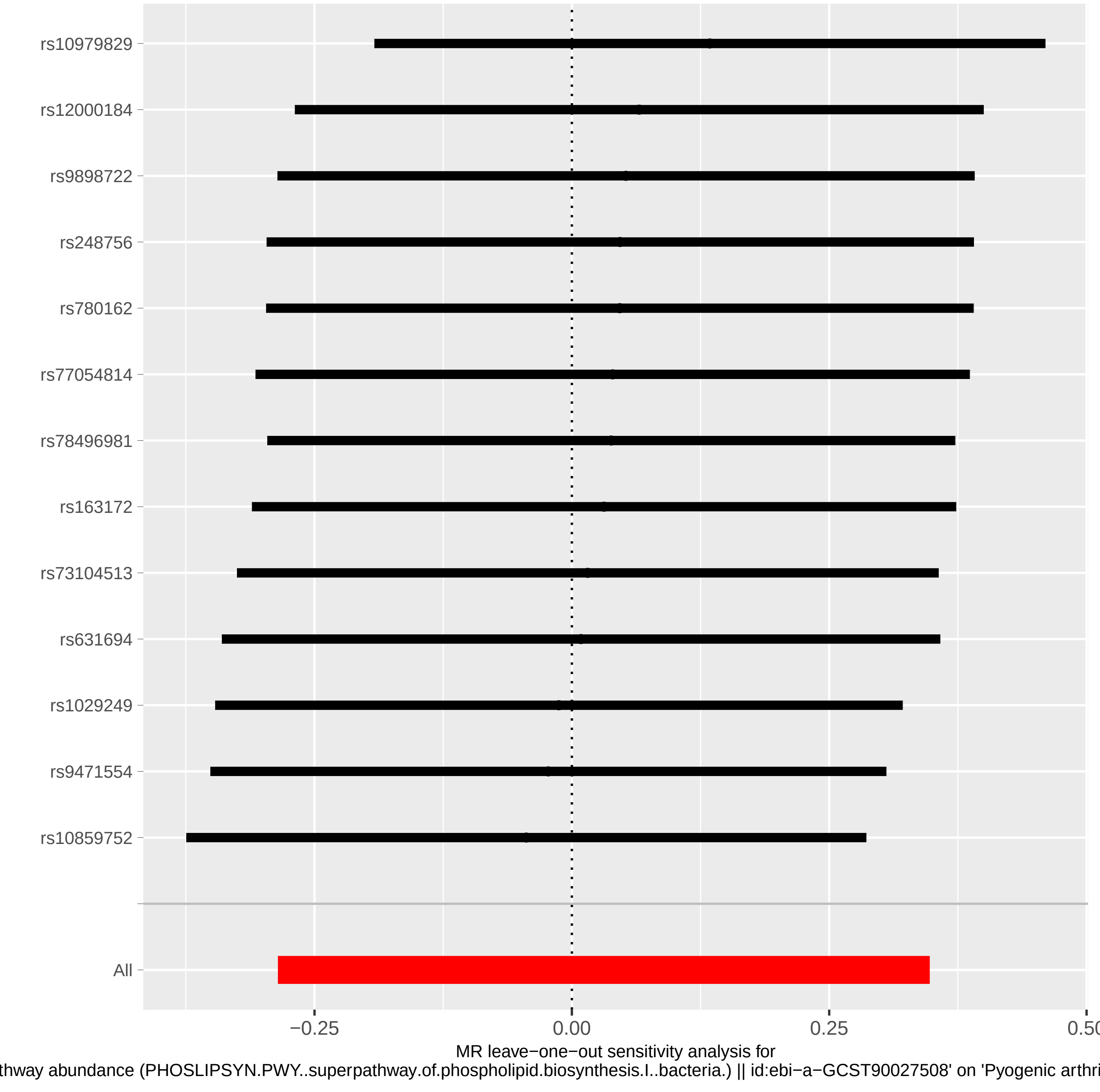
rs10979829
rs12000184
rs9898722
rs248756
rs780162
rs77054814
rs78496981
rs163172
rs73104513
rs631694
rs1029249
rs9471554
rs10859752
All
−0.25
0.00
0.25
0.50
MR leave−one−out sensitivity analysis for
thway abundance (PHOSLIPSYN.PWY..superpathway.of.phospholipid.biosynthesis.I..bacteria.) || id:ebi−a−GCST90027508' on 'Pyogenic arthri
